# 

**Published:** 1897-05-22

**Authors:** 


### EARLY EDITION.

**Notices of Births, Deaths, and Marriages** are inserted in this column at a charge of 2s. 6d. for an announcement not exceeding four lines.

### NOTICE TO CORRESPONDENTS.

All MS., Letters, Books for Review, and other matters intended for the Editor should be addressed The Editor, "The Hospital," The Hospital Building, 28 & 29, Southampton Street, Strand, W.C.

The Editor cannot undertake to return rejected MS., even when accompanied by stamped directed envelope.

**Notice to Advertisers and Subscribers.**

All Advertisements, Orders for Copies of The Hospital, and Business Communications should be addressed to **THE MANAGER, (Not to The Editor),** The Hospital, The Hospital Building, 28 & 29, Southampton Street, Strand, London, W.C.

The Cost of Subscription, post free, is as follows:—Per week, 2½d.; per quarter, 2s. 8d.; per half-year, 5s. 3d.; per annum, 10s. 6d. Foreign:—Per annum, 15s. 2d.; payable, in all cases, in advance.

**NOTICE.—Vol. XXI. of "The Hospital" (October, 1896, to March, 1897), in handsome cloth binding, is on sale at the Office of this Journal, price 7s. 6d. Cases for binding the Numbers contained in this Volume, 1s. 6d. Index to Vol. XXI., 2½d., post free.**

**The Monthly Part for June will be ready on June 1st. Price 6d., by post 8d.**

### BOOKS RECEIVED.

J. B. Lippincott Company.

"Genito-Urinary Surgery and Venereal Diseases." By J. William White, M.D., and Edward Martin, M.D.

J. and A. Churchill.

"A Short Practice of Midwifery." By Henry Jellett, M.D., with a preface by W. J. Smyly, M.D.

Isbister and Co.

"Poems Dedicated to National Independence and Liberty." By William Wordsworth, with an introduction by Stopford A. Brooke.

"Salisbury Cathedral." By the Very Rev. G. D. Boyle, M.A. Illustrated by Alexander Ansted.

"Gloucester Cathedral." By the Very Rev. Dean Spence. Illustrated by Herbert Railton.

"Canterbury Cathedral." By the Very Rev. Dean Freemantle. Illustrated by W. Lapworth.

"Norwich Cathedral." By the Very Rev. Dean Lefroy. Illustrated by Alexander Ansted.

The "Lancet" Office.

"Quarantine in England." By William Collingridge, M.A., M.D.

Whitley and Booth.

"Report of the Medical Officer, County Borough of Halifax."

Cassell and Co.

"London Health Laws Issued by the Mansion House Council on the Dwellings of the Poor."

**Periodicals and Pamphlets Received.**—Knowledge, The Zenana, Columbus Medical Journal, The Woman's Signal, Medical Press, Literary Digest, Nursing Notes, Industries and Iron, Leeds Hospital Magazine, Charity Organisation Review, London, New York Medica Record, Electrical Review, Girl's Own Paper, Boy's Own Paper, Sunday at Home, Leisure Hour, Sunday Hours, Boy's Sunday Monthly, The Wombat, Institutional Buildings Completed or Commenced During the Diamond Jubilee of Queen Victoria by J. Passmore Edwards.

